# Membranous nephropathy and autoimmune hepatitis in the setting of acute *Helicobacter pylori* infection: a case report

**DOI:** 10.1186/s13256-021-02874-7

**Published:** 2021-05-30

**Authors:** Zeid Nesheiwat, Judy Daboul, Ganesh Prasad Merugu, Sreedhar Adapa, Mamtha Balla

**Affiliations:** 1 Department of Internal Medicine, The University of Toledo College of Medicine, 2100 Central Avenue 2nd floor, Toledo, Ohio 43606 USA; 2 Division Chief and Geriatric Medicine Fellowship Director, Division of Geriatric Medicine, Department of Family Medicine, The University of Toledo College of Medicine, 3000 Arlington Avenue, Toledo, Ohio 43614 USA; 3 Department of Internal Medicine, Division of Nephrology, Adventist Medical Center, 115 Mall Drive, Hanford, CA 93230 USA; 4 The University of Toledo College of Medicine, ProMedica Physician Hospitalist, ProMedica Toledo Hospital, 2142 N Cove Blvd, Toledo, Ohio 43606 USA; 5 Department of Internal Medicine, The University of Toledo College of Medicine, 3000 Arlington Avenue, Toledo, Ohio 42614 USA

**Keywords:** Membranous nephropathy, *Helicobacter pylori* infection, Nephrotic syndrome, Autoimmune diseases, Hepatitis, Proteinuria, Kidney biopsy, Case report

## Abstract

**Background:**

Membranous nephropathy (MN) is the leading cause of nephrotic syndrome in adults worldwide. A growing body of evidence indicates a pathogenic and autoimmune correlation between *Helicobacter pylori* infection, MN, and autoimmune liver disease.

**Case presentation:**

A 47-year-old African American woman presented to our institution with epigastric pain and vomiting. In-patient hospital workup included a thorough abdominal evaluation including esophagogastroduodenoscopy and liver biopsy, which revealed active *H. pylori* infection and autoimmune hepatitis. The patient was incidentally also found to have nephrotic-range proteinuria. Renal workup including kidney biopsy established the diagnosis of MN. Proteinuria improved after initiation of triple therapy for *H. pylori* infection.

**Conclusion:**

This case adds to the growing evidence of a correlation between *H. pylori* infection, MN, and autoimmune liver disease. This report demonstrates a unique case of a patient with MN, autoimmune hepatitis (AIH)/primary biliary cholangitis (PBC), and HP who underwent triple-eradication antibiotic treatment that resulted in an ultimate resolution of all these conditions.

## Background

Membranous nephropathy (MN) is a leading cause of nephrotic syndrome in adults worldwide [1]. It is most commonly idiopathic; however, it can be secondary to autoimmune disease, neoplasia, infection, drugs, and others [2]. Increasingly, many reports indicate a pathogenic correlation between *Helicobacter pylori* (HP) infection and MN [3]. Indeed, HP antigens have been found in the glomeruli of MN patients, and eradication of HP successfully reduced proteinuria [3, 4]. Although most reported occurrences have been seen in diabetics, a relationship in non-diabetics does exist [3, 5]. Several other antigens such as hepatitis B and hepatitis C have also been associated with secondary MN [6]. We report the case of an adult nondiabetic black female patient who presented with abdominal pain and nausea and was found to have gastric ulcers positive for HP in conjunction with autoimmune hepatitis (AIH) and MN.

## Case presentation

A 47-year-old African American woman with a past medical history of essential hypertension, hyperlipidemia, benign gastric tumors status post-resection in 2008, and nicotine dependence presented to our institution with a chief complaint of epigastric pain and vomiting for 3 weeks. The patient’s home medications included cholecalciferol (D3) 1000 units per day and a daily multivitamin. She also reported loss of appetite and dark-colored urine. Initial vital signs were stable, with blood pressure of 150/65 mmHg, temperature of 99.1 °F, heart rate of 66 beats per minute, respiratory rate of 22 breaths/minute, and oxygen saturation of 100% on room air. Physical exam revealed abdominal tenderness with a positive Murphy’s sign. Additional physical exam findings showed bilateral lower extremity edema, supple neck with a non-enlarged and non-nodular thyroid, and no evidence of arthritis or erythematous joints. The rest of the physical examination was unremarkable.

The laboratory workup is shown in Table 1. Pertinent lab values include elevated alkaline phosphatase (688 U/L), aspartate transaminase (134 U/L), alanine transaminase (136 U/L), total bilirubin (2.9 mg/dL), and direct bilirubin (1.8 mg/dL), with borderline hemoglobin (11.4 g/dL) and albumin (3.2 g/dL), with normal renal function. Other lab work including urinalysis with microscopy revealed high protein > 1000 mg/dL, glucose 100 mg/dL, ketones 80 mg/dL, urobilinogen 4.0 mg/dL, high bilirubin, red blood cells (RBC) 50/high-power field (HPF), squamous epithelium 7/HPF, cellular cast < 1, hyaline casts 22/HPF, granular casts 2, and white blood cells (WBC) 4/HPF. A right upper quadrant ultrasound showed sludge in the mid- and proximal common bile duct (CBD), with no dilation. There was also a 1.1 × 0.9 × 1.3 cm echogenic lesion within the right lobe of the liver, which was described as likely a benign hemangioma. Other findings were heterogeneous echotexture of the liver with echogenic portal triads suggestive of an inflammatory process including hepatitis. In addition, abdominal magnetic resonance imaging (MRI) was negative for any lesions corresponding to the 1.1 cm echogenic hepatic lesion seen on ultrasound, suggesting likely transient fatty liver infiltration. Gastroenterology and nephrology were consulted for further management.

**Table 1:** Laboratory values at initial presentation

	Reference range	Results
White blood cell (WBC)	4.8–10.8 × 10^−9^/L	8.1
Hemoglobin	11.7–16.0 g/dL	11.4
Hematocrit	35–47 %	34.0
Mean corpuscular volume	81–100 fL	84
Platelets	150–450 × 10^−9^/L	302
Sodium	134–146 mmol/L	133
Potassium	3.5–5 mmol/L	3.3
Creatinine	0.40–1.00 mg/dL	0.53
Blood urea nitrogen (BUN)	5–23 mg/dL	10
Bicarbonate (CO^2^)	22–32 mmol/L	26
Anion gap	4–12 mmol/L	9
Glomerular filtration rate (GFR)	> 59 mL/min/1.73sq.m	> 60
Alkaline phosphatase	39–130 U/L	688
Aspartate transaminase (AST)	0–41 U/L	134
Alanine transaminase (ALT)	0–31 U/L	136
Total bilirubin	0.3–1.2 mg/dL	2.9
Bilirubin, direct	0.0–0.4 mg/dL	1.8
Albumin	3.2–5.3 g/dL	3.2
Total protein	6.0–8.0 g/dL	8.1
Lipase	11–82 U/L	47
Urinalysis with microscopy		
Protein	Negative	> 1000
Glucose	Negative	Negative
Ketones	Negative	80
Urobilinogen	< 1.1 mg/dL	4
Bilirubin	Negative	Large
Red blood cells (RBC)	0–5/HPF	50
White blood cells (WBC)	0–5/HPF	4
Squamous epithelium	0–5/HPF	7
Cellular casts	0/LPF	< 1
Hyaline casts	0–2/LPF	22
Granular casts	0/LPF	2

*HPF* high-power field, *LPF* low-power field

**Fig. 1:**
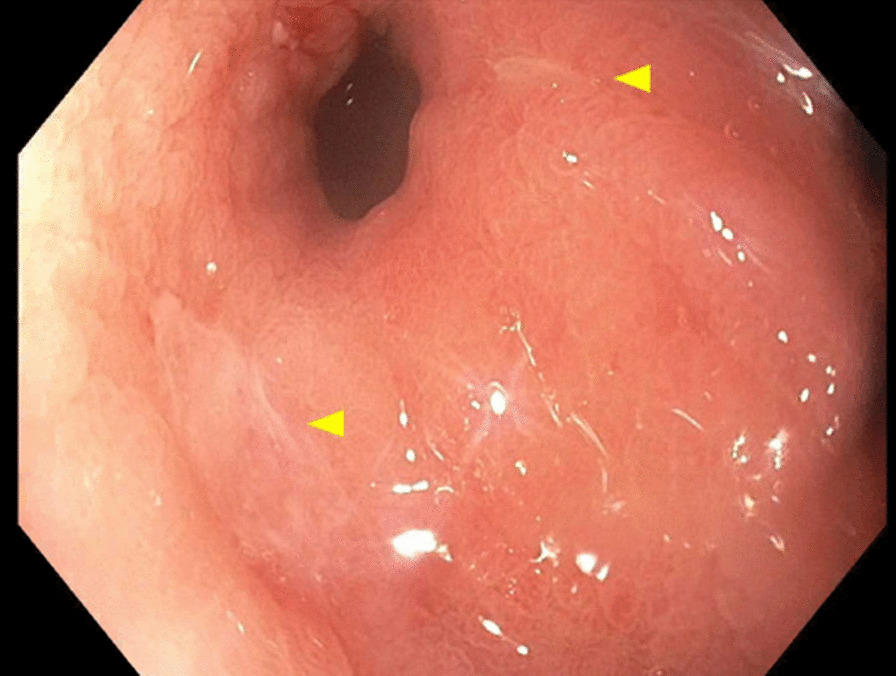
Esophagogastroduodenoscopy showing non-bleeding ulcers in prepyloric regions of the stomach (yellow arrows)

**Fig. 2:**
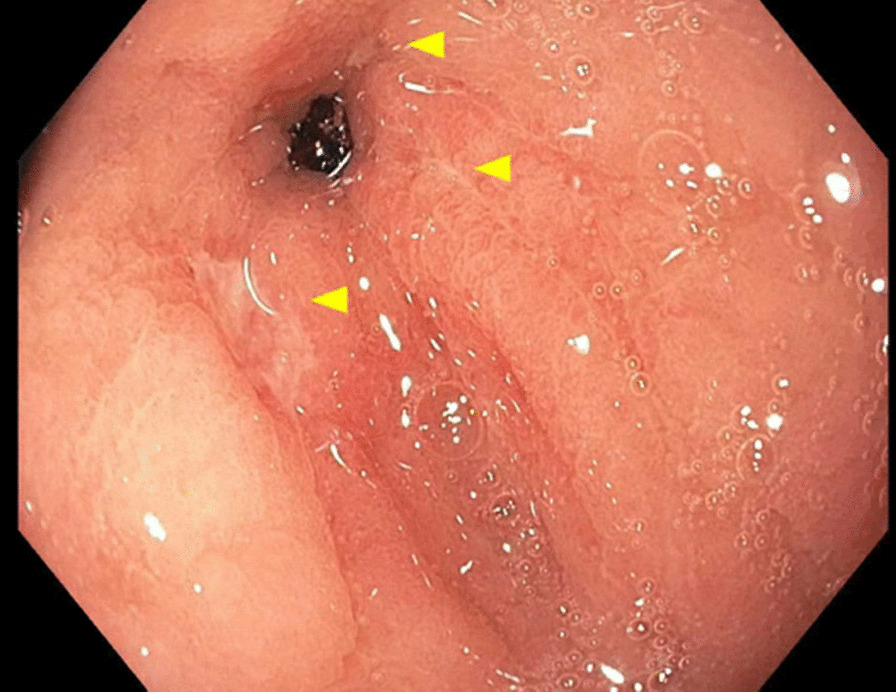
Esophagogastroduodenoscopy showing non-bleeding ulcers in prepyloric regions of the stomach (yellow arrows)

Initial gastroenterology workup revealed negative results for a hepatitis panel which included hepatitis A immunoglobulin M (IgM) antibody, hepatitis B surface antigen, hepatitis B core IgM antibody, and anti-hepatitis C with reflex polymerase chain reaction. Iron studies revealed anemia of chronic disease with a low iron level of 32 µg/dL, low total iron-binding capacity (TIBC) 168 µg/dL, normal ferritin 304 ng/mL, and iron saturation of 19%. Additional lab work was positive for anti-mitochondrial antibody (1:80 titer) and anti-smooth muscle antibody (1:80 titer). The patient underwent esophagogastroduodenoscopy (EGD) for persistent nausea and vomiting that showed inflammation in the gastric body/antrum, three non-bleeding ulcers in prepyloric regions of the stomach (largest lesion 9 mm), and biopsy positive for HP (Figs. 1 and 2). The patient was started on triple therapy for her HP infection (amoxicillin 1 g twice daily, clarithromycin 500 mg twice daily, and pantoprazole 40 mg twice daily for 14 days).

**Fig. 3:**
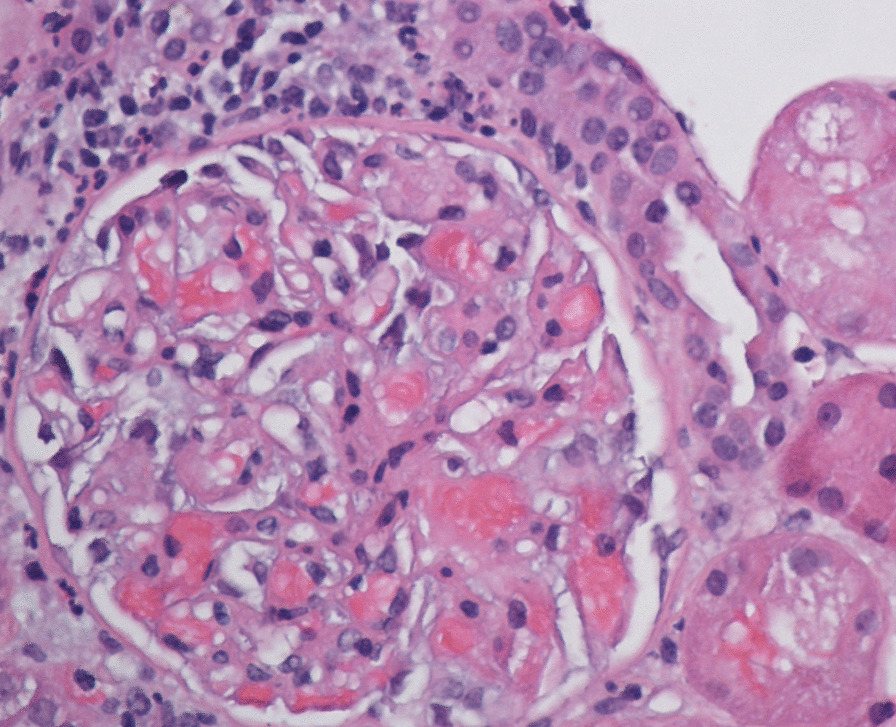
Hematoxylin and eosin stain (×40) shows normal glomerulus in size and cellularity with thickening of glomerular basement membranes

**Fig. 4.**
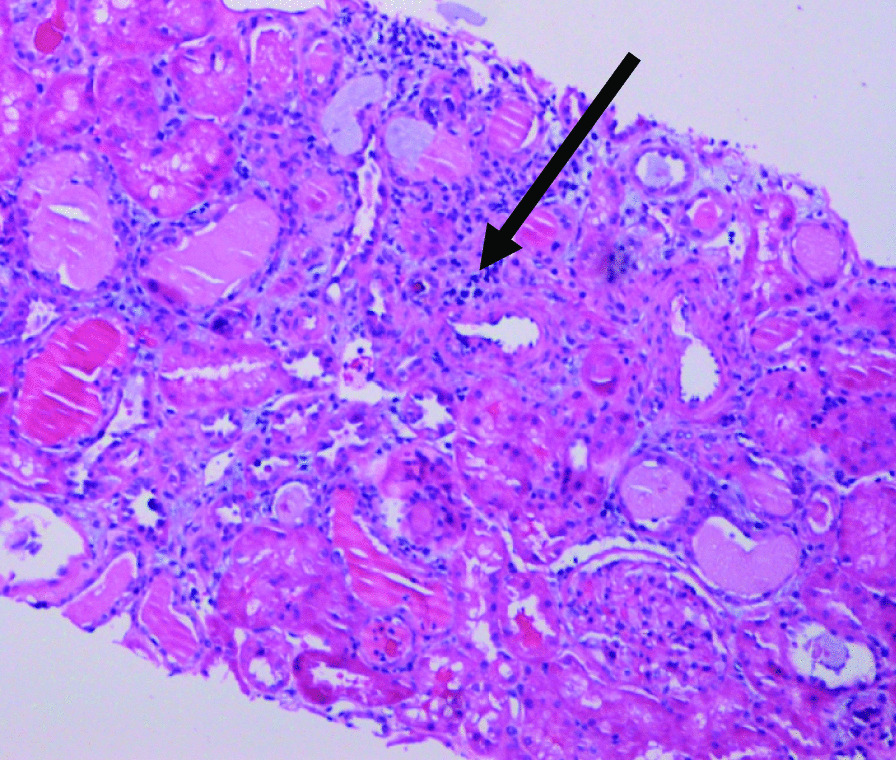
Hematoxylin and eosin stain (×20) shows many hyaline casts filled the proximal and distal tubules with mild focal interstitial inflammation (black arrow)

**Fig. 5:**
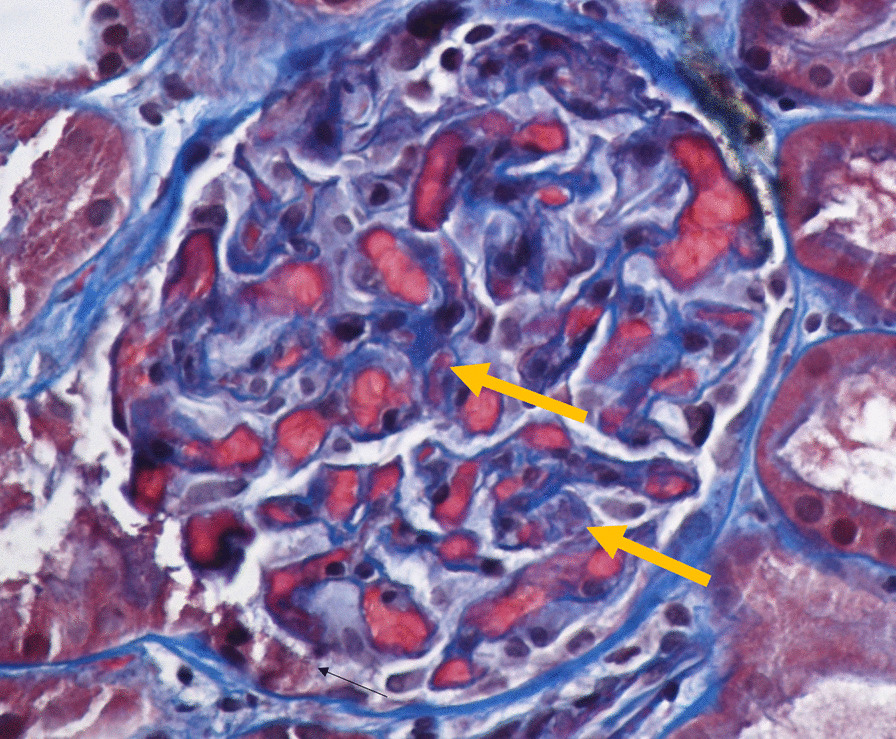
Masson trichrome stain (×40 shows red subepithelial deposits (yellow arrows)

**Fig. 6:**
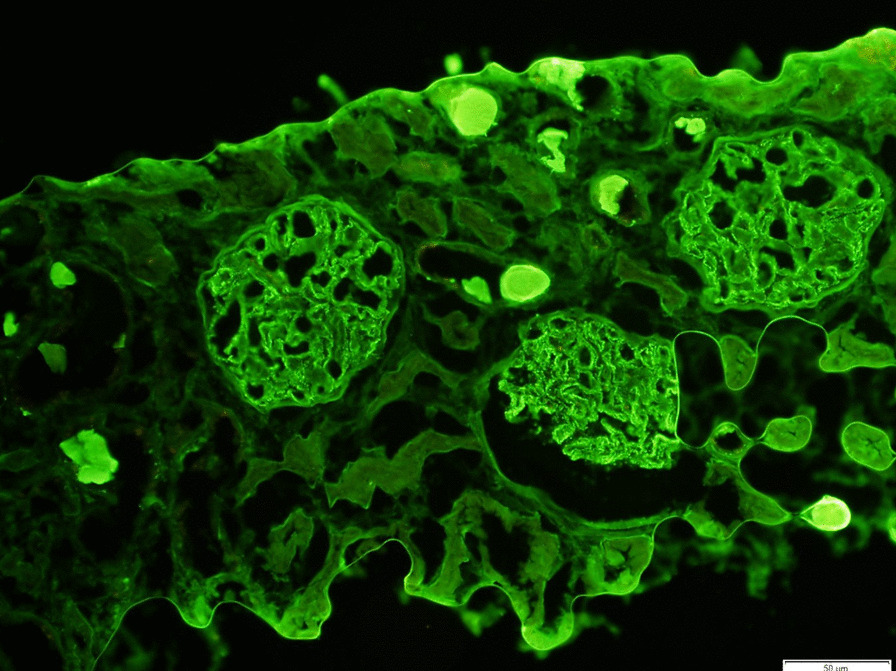
Immunofluorescence staining for immunoglobulin G (IgG) shows 2+ granular global capillary wall positivity for IgG

**Fig. 7:**
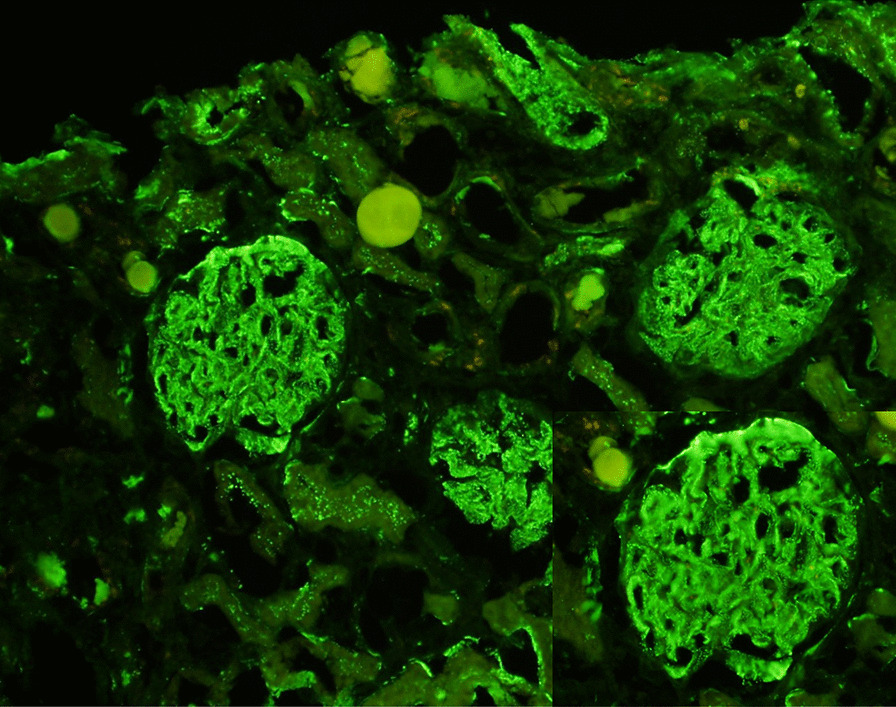
Immunofluorescence staining for complement C3 shows 3+ diffuse positivity for C3

**Fig. 8:**
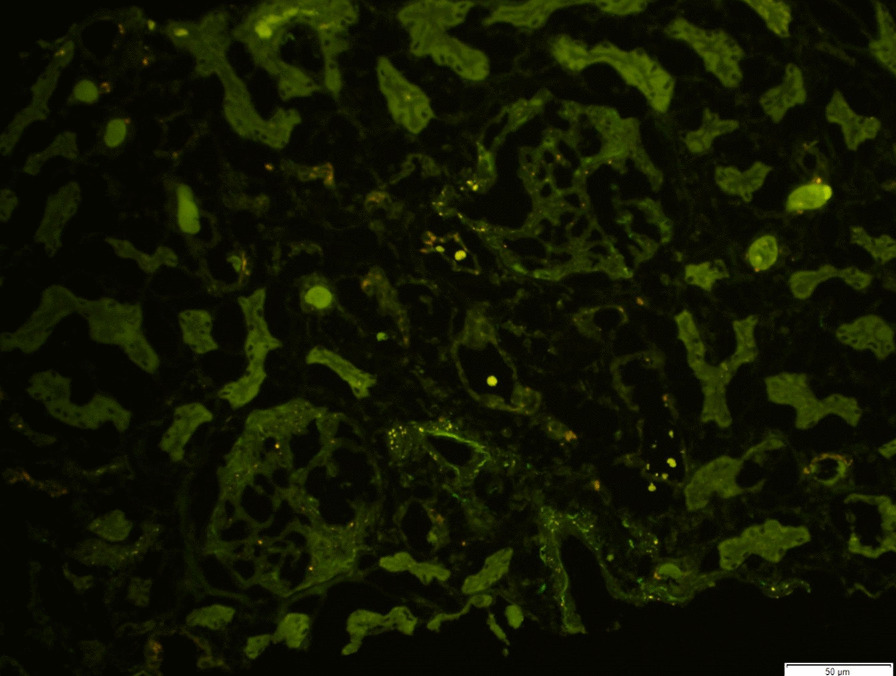
Immunofluorescence staining for antiphospholipid A2 receptor (PLA2R) shows negative immunofluorescence

**Fig. 9:**
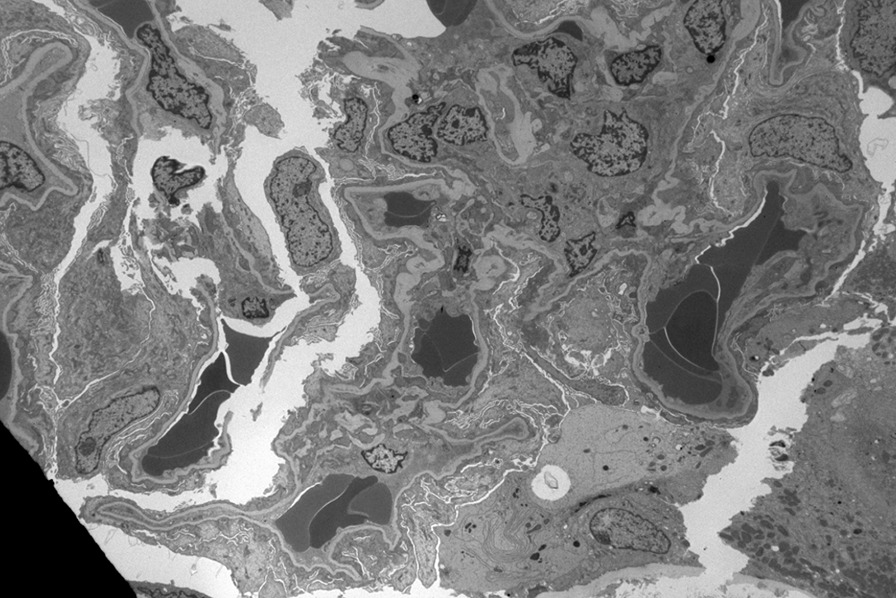
Electron microscopy shows global effacement of podocyte foot processes

**Fig. 10:**
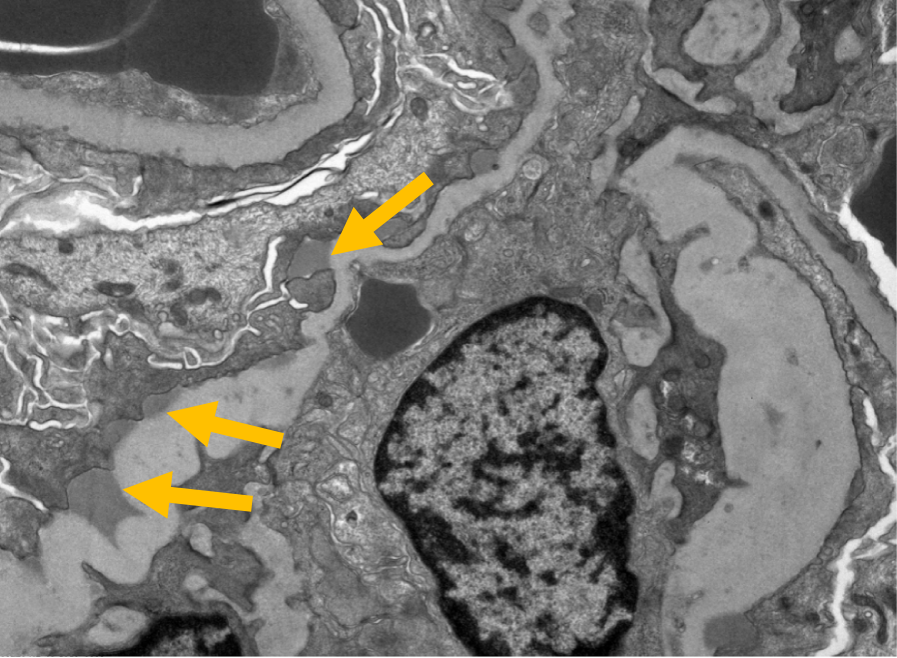
(Electron microscopy shows small to medium-sized subepithelial electron-dense immune complex deposits (yellow arrows)

Nephrology workup simultaneously showed a urine protein-to-creatinine ratio of 35.7 mg/mg and subsequent 24-hour protein excretion of 35,088 mg/24 hours, signifying nephrotic-range proteinuria. An ultrasound of the kidney revealed no hydronephrosis or acute pathology. The serological workup was negative, including antinuclear antibody (ANA), double-stranded DNA (dsDNA), complement, serum and urine electrophoresis with immunofixation, free light chain ratio, human immunodeficiency virus (HIV) titer, antineutrophil cytoplasmic antibody (ANCA), anti-glomerular basement membrane antibody titers, and antiphospholipid A2 receptor (PLA2R), and the patient subsequently underwent renal biopsy, which revealed MN. Hematoxylin and eosin (H&E), periodic acid–Schiff (PAS), Masson trichome, and Jones methenamine silver were used to stain the tissues. Light microscopy revealed thickening of the glomerular basement membrane, and on trichrome stain, red subepithelial deposits were seen. There was also mild patchy interstitial edema and focal inflammation composed mainly of lymphocytes and monocytes. Immunofluorescence histology showed 2+ granular global glomerular positivity for immunoglobulin G (IgG) and negative phospholipase A2 receptor (PLA2R). The renal biopsy images are shown in Figs. 3, 4, 5, 6, 7, 8, 9, and 10. The patient was administered bumetanide 1 mg twice daily as diuretic for bilateral lower extremity swelling and started on losartan 50 mg daily for proteinuria suppression and renal protection.

In addition, the patient underwent laparoscopic cholecystectomy for biliary colic with liver biopsy during her hospital stay. The patient’s proteinuria continued to improve despite not using immunosuppressive therapy and coincided with her HP treatment. The patient was then discharged with gastroenterology and nephrology follow-up.

At the patient’s first gastroenterology outpatient visit, the liver biopsy showed chronic hepatitis with portal, periportal, and focal septal fibrosis (fibrosis stage II) and increased iron deposits involving Kupffer cells (grade 2). The patient was started on prednisone 40 mg daily and azathioprine for AIH. The patient’s proteinuria had been improving before initiation of immunosuppressive treatment. Table 2 shows a timeline of pertinent events.

**Table 2:** Timeline of pertinent events

October 26, 2019	Patient admitted for nausea and vomiting; urine protein 38,280 mg/L incidentally found
October 29, 2019	Esophagogastroduodenoscopy performed and *Helicobacter pylori* treatment initiated
October 31, 2019	Laparoscopic cholecystectomy and liver biopsy
November 3, 2019	Re-measured urine protein 4730 mg/L
November 4, 2019	Renal biopsy performed
November 5, 2019	Patient discharged. Losartan was started
November 6, 2019	Liver biopsy results come back revealing autoimmune hepatitis
November 11, 2019	Prednisone started for autoimmune hepatitis
November 13, 2019	Renal biopsy results showing membranous nephropathy
December 3, 2019	Azathioprine started after thiopurine methyltransferase genetic testing

## Discussion and conclusion

MN is among the most common causes of nephrotic syndrome in adults, accounting for approximately one third of all cases. It is especially prevalent in Caucasian adults, with a 2:1 male preponderance, and a peak incidence in individuals aged 50–60 years [7].

Nephrotic syndrome is defined as 24-hour urine protein > 3.5 g/day, hypoalbuminemia < 3 g/dL, peripheral edema, and hyperlipidemia [8]. There is increasing evidence for an autoimmune basis in idiopathic MN. This has been supported by certain target antigens on the surface of podocytes that have been recognized in the vast majority of primary MN, the M-type phospholipase A2 receptor 1 (PLA2R), and to a much lesser extent the thrombospondin type 1 domain-containing 7A [9]. PLA2R autoantibodies have been found in the serum in the majority (52–82%) of patients with primary MN and are typically absent in patients with secondary MN conditions [10]. Our patient tested negative for PLA2R, indicating a possible secondary pathogenesis contributing to the MN; however, primary MN is still possible. Thrombospondin was not tested and can be considered a limitation in our report. Furthermore, multiple studies have found autoantibodies to podocyte antigens, predominantly belonging to the IgG4 subclass; these podocyte-specific autoantibodies can be present in both the serum and glomeruli of patients [11]. Our patient had capillary wall positivity for IgG1, IgG2, and IgG3, and only trace IgG4. In one case series, PLA2R autoantibodies were characteristically absent in IgG4-related MN; however, other studies have found coexistence of PLA2R and IgG4 [12, 13]. While autoimmune diseases such as systemic lupus erythematous have long been associated with MN, more recently other vague autoimmune syndromes, including inflammatory arthritis and idiopathic thrombocytopenia purpura, have also been reported [14].

In this report, we present the case of a nondiabetic black woman with extremely high proteinuria of approximately 35 g of protein excreted in 24 hours; she was incidentally found to have MN following AIH and PBC. The patient was found to have positive anti-smooth muscle antibody as well as positive anti-mitochondrial antibody and high IgG levels, supporting the diagnosis of AIH, and suggesting concomitant primary biliary cholangitis (PBC) as well. This finding is particularly interesting because it seems to support the rising autoimmune basis behind MN, especially in conjunction with the high IgG levels. Another possible mechanism involves the binding of circulating antibodies to intrinsic glomerular antigens [7, 15]. Similarly, Bindi *et al*. extracted anti-mitochondrial globulins from kidney epi-membranous deposits, which provides evidence of a common immune mechanism between PBC and MN [16]. A few cases have been previously reported linking MN with PBC or AIH, and even linking MN with both AIH and PBC [13, 17]. Nevertheless, an underlying mechanism remains to be elucidated. Furthermore, in one case study of a patient with AIH and PBC overlapping with MN, treatment with prednisone, azathioprine, and ursodeoxycholic acid returned liver function tests to normal after 1 month, as well as decreasing urinary protein excretion [17]. The patient in this case study underwent similar treatment with prednisone and azathioprine, with complete resolution of liver transaminitis and urine protein excretion.

The present case was further complicated by the finding of several gastric ulcers positive for HP at the time of diagnosis of PBC, AIH, and MN. Multiple reports have noted an association between HP and MN. In one study we reviewed, a higher incidence of HP was found in patients with MN than age-matched control subjects [18]. Furthermore, triple therapy for HP in patients with MN was found to be correlated with a significant decrease in proteinuria, although whether that is in direct response or due to spontaneous remission of MN is unclear [3]. Interestingly, HP has also been proposed in the pathogenesis of PBC as well as other autoimmune liver conditions. More patients tested positive for multiple antibodies including anti-mitochondrial antibody and anti-smooth muscle antibody, among others, in patients with HP than in patients without HP with coexisting AIH [19]. Furthermore, in one report of a case including celiac disease, PBC, and HP, eradication of HP and treatment with ursodeoxycholic acid led to improvement of PBC. This suggests a hypothesis of microbial exposure leading to antibody response by a cross-reactive mechanism [20]. An alternative hypothesis is that patients with MN are more prone to HP infection, as hypoalbuminemia may predispose patients to becoming immunocompromised [3]. This patient also underwent triple therapy for HP with eventual resolution of MN and transaminitis, as well as resolution of HP as evidenced by a negative breath test. However, it is unclear which therapy was the most effective in the treatment of MN.

MN is a multifaceted cause of nephrotic syndrome in adults and has a strong autoimmune pathogenesis. There is an association between MN and AIH/PBC which may suggest the coincidental presence of multiple autoimmune conditions or a possible mechanism in which one contributes to the formation of the other. HP is also an interesting microbial condition associated with many autoimmune conditions such as MN and AIH/PBC, and many hypotheses on the pathogenesis thereof have been proposed. This case report demonstrated a unique case of a patient with MN, AIH/PBC, and HP who underwent triple-eradication antibiotic treatment as well as prednisone and azathioprine, resulting in an ultimate resolution of all these conditions.

## Data Availability

Not applicable.

## Abbreviations

MN: Membranous nephropathy
AIH: Autoimmune hepatitis
PBC: Primary biliary cholangitis
HP: *Helicobacter pylori*
RBC: Red blood cell
CBD: Common bile duct
T2DM: Type II diabetes mellitus
